# Pre-hospital CPR and early REBOA in trauma patients — results from the ABOTrauma Registry

**DOI:** 10.1186/s13017-020-00301-8

**Published:** 2020-03-30

**Authors:** Peter Hilbert-Carius, David T. McGreevy, Fikri M. Abu-Zidan, Tal M. Hörer, and the ABOTrauma Registry research group, D. T. McGreevy, P. Hilbert-Carius, F. M. Abu-Zidan, T. M. Hörer, M. Sadeghi, A. Pirouzram, A. Toivola, P. Skoog, K. Idoguchi, Y. Kon, T. Ishida, Y. Matsumura, J. Matsumoto, M. Maszkowski, A. Bersztel, E. C. Caragounis, M. Falkenberg, L. Handolin, S. W. Chang, B. Kessel, D. Hebron, G. Shaked, M. Bala, F. Coccolini, L. Ansaloni, T. Larzon, K. F. Nilsson

**Affiliations:** 1 Department of Anesthesiology, Emergency and Intensive Care Medicine, Bergmannstrost BG-Klinikum Halle gGmbH, Merseburgerstr. 165, 06112 Halle, Germany; 2 Department of Cardiothoracic and Vascular Surgery, Faculty of Medicine and Health, Örebro University, Örebro, Sweden; 3 Department of Surgery, College of Medicine and Health Science, UAE University, Al-Ain, United Arab Emirates

**Keywords:** REBOA, Cardiac arrest, Trauma, CPR, Endovascular resuscitation

## Abstract

**Background:**

Severely injured trauma patients suffering from traumatic cardiac arrest (TCA) and requiring cardiopulmonary resuscitation (CPR) rarely survive. The role of resuscitative endovascular balloon occlusion of the aorta (REBOA) performed early after hospital admission in patients with TCA is not well-defined. As the use of REBOA increases, there is great interest in knowing if there is a survival benefit related to the early use of REBOA after TCA. Using data from the ABOTrauma Registry, we aimed to study the role of REBOA used early after hospital admission in trauma patients who required pre-hospital CPR.

**Methods:**

Retrospective and prospective data on the use of REBOA were collected from the ABOTrauma Registry from 11 centers in seven countries globally between 2014 and 2019. In all patients with pre-hospital TCA, the predicted probability of survival, calculated with the Revised Injury Severity Classification II (RISC II), was compared with the observed survival rate.

**Results:**

Of 213 patients in the ABOTrauma Registry, 26 patients (12.2%) who had received pre-hospital CPR were identified. The median (range) Injury Severity Score (ISS) was 45.5 (25–75). Fourteen patients (54%) had been admitted to the hospital with ongoing CPR. Nine patients (35%) died within the first 24 h, while seventeen patients (65%) survived post 24 h. The survival rate to hospital discharge was 27% (*n* = 7). The predicted mortality using the RISC II was 0.977 (25 out of 26). The observed mortality (19 out of 26) was significantly lower than the predicted mortality (*p* = 0.049). Patients not responding to REBOA were more likely to die. Only one (10%) out of 10 non-responders survived. The survival rate in the 16 patients responding to REBOA was 37.5% (*n* = 6). REBOA with a median (range) duration of 45 (8–70) minutes significantly increases blood pressure from the median (range) 56.5 (0–147) to 90 (0–200) mmHg.

**Conclusions:**

Mortality in patients suffering from TCA and receiving REBOA early after hospital admission is significantly lower than predicted by the RISC II. REBOA may improve survival after TCA. The use of REBOA in these patients should be further investigated.

## Background

Globally, trauma with massive bleeding is the second leading cause of death under the age of 40 years [1]. Traumatic cardiac arrest (TCA) has an extremely high mortality especially in blunt trauma. Cardiopulmonary resuscitation (CPR) after trauma is considered to be of little benefit [2]. CPR is initiated when the carotid pulse cannot be palpated in an unresponsive patient. However, this does not per se confirm “true” cardiac arrest but may represent a state of inadequate perfusion with impending cardiac arrest. A current study demonstrated that REBOA in patients with impending cardiac arrest is feasible and showed a survival rate of 37% [3]*.* The international resuscitation guidelines (ERC and AHA) advocate a consistent approach to CPR on the basis of up-to-date evidence and expert’s consensus opinions about the use of invasive measures, including resuscitative thoracotomy (RT), in order to eliminate reversible causes of cardiac arrest in trauma patients [2, 4–6]. One invasive measure to prevent patients from exsanguination in non-compressible torso hemorrhage, but not yet mentioned in the CPR guidelines, is resuscitative endovascular balloon occlusion of the aorta (REBOA) [7, 8]. REBOA may have the potential to temporarily diminish exsanguination at the expense of ischemia [9]. The role of early REBOA after hospital admission in patients with pre-hospital TCA and consecutive CPR, either arriving with a return of spontaneous circulation (ROSC) or ongoing CPR, is not yet clear. The possible control of bleeding and the hemodynamic effect of REBOA with improvement in coronary and cerebral perfusion pressure may have a positive survival benefit [10, 11]. On the other hand, REBOA serves as a bridge to definitive surgical bleeding control and treatment and is therefore a procedure used to gain time and not for definitive care [12].

Surprisingly, recently published registry-based work by Joseph et al. did not find a positive survival benefit for patients who were treated with REBOA in the American College of Surgeons Trauma Quality Improvement Program data set [13]. Using the ABOTrauma Registry, we aimed to study the role of early REBOA on arrival to the hospital in trauma patients who had received pre-hospital CPR due to TCA.

## Patients and methods

Data from the ABOTrauma Registry between 2014 and 2019 were analyzed. The ABO (aortic balloon occlusion) Trauma Registry is a registry that only collects data from trauma patients in whom REBOA was deployed. Trauma patients receiving REBOA for treatment of hemorrhagic shock at 11 centers from seven countries were included. The ABOTrauma Registry provides retrospective and prospective data for trauma patients in hemorrhagic shock in whom REBOA had been used. Center recruitment is ad hoc, with known REBOA-practicing institutions invited to participate directly. Centers can also register independently via the registry website after approval from the principal investigators. To capture clinically pragmatic data, there are no center-specific criteria such as minimum case volume or hospital size. The registry is funded and hosted by the Department of Cardiothoracic and Vascular Surgery, Örebro University Hospital, Sweden. Ethical approval for the registry was obtained from the regional committee (study number: 2014/210; Regionala Etikprövningsnämnden, Uppsala, Sweden). Patient data are anonymized at the point of registration with a unique registry-generated ID number. No patient identifiable data (name, hospital number, date of birth) are held in the registry, and all data are held on a secure electronic database. A secured password has been given to centers joining the registry to be able to enter data, and the registry is in line with the current European data protection regulation. The need for ethical approval of the current study was waived by the ethical committee of the Medical Association Saxony-Anhalt Germany.

Inclusion criteria for the present study were pre-hospital CPR initiated due to TCA and having complete data to calculate the probability of survival using the revised injury severity classification II (RISC II), availability of outcome data (return of spontaneous circulation - ROSC, survival, death), and REBOA (including REBOA-Zone) performed early after admission. TCA was defined as a non-palpable pulse on a large central artery (carotid or common femoral artery) with necessary CPR due to trauma. ROSC was defined as a palpable pulse in the mentioned arteries with no further need for external cardiac compression.

Exclusion criteria were no pre-hospital CPR and missing data regarding the outcome or RISC II calculation.

The RISC score has been developed using data from the German Trauma Registry [14]. The update of the Revised Injury Severity Classification score, the RISC II, has been developed using 30,866 patients and was validated with 21,918 patients [15]. Our opinion it is the best trauma score for predicting outcomes currently available. The following variables were used to predict survival: New Injury Severity Score (NISS), age, head injury, Glasgow Coma Scale (GCS), coagulation (partial thromboplastin time), base deficit, CPR (pre-hospital or after admission), and number of indirect signs of bleeding (low hemoglobin, hypotension, and massive transfusion). For most variables, an algorithm for replacing missing values had been established. The RISC II includes several new predictors, such as pupil size and reactivity, but also an innovative type of management of missing values [15]. The probability of survival is calculated using the logistic function:

$$ P\left(\mathrm{survival}\right)=\frac{1}{1+\exp \left(-X\right)} $$

*X* is the logit or natural logarithm of the odds of the depending variable (see above) occurring or not [15]. The RISC II and the score model are detailed elsewhere [15].

## Data analysis

Continuous or ordinal data were reported as median (range) while categorical data were reported as numbers (percentages). If data were missing, valid percentages were calculated from the available data. Non-parametric statistical methods were used due to the sample size of the groups. These methods do not need a normal distribution because they analyze the ranks and not the crude number. Mann-Whitney *U* test was used to compare ordinal or continuous data for two independent groups. Fisher’s exact test was used to compare categorical data of two independent groups, and Wilcoxon signed test was used to compare continuous data for two dependent groups. The standardized mortality ratio (SMR), calculated by dividing the observed mortality by the predicted mortality using the RISC II, was calculated to show any outcome effect. Statistical analysis was performed using SPSS version 20 (IBM, Armonk, NY, USA). Statistical significance was set at *p* ≤ 0.05 (two-tailed).

## Results

**Fig. 1:**
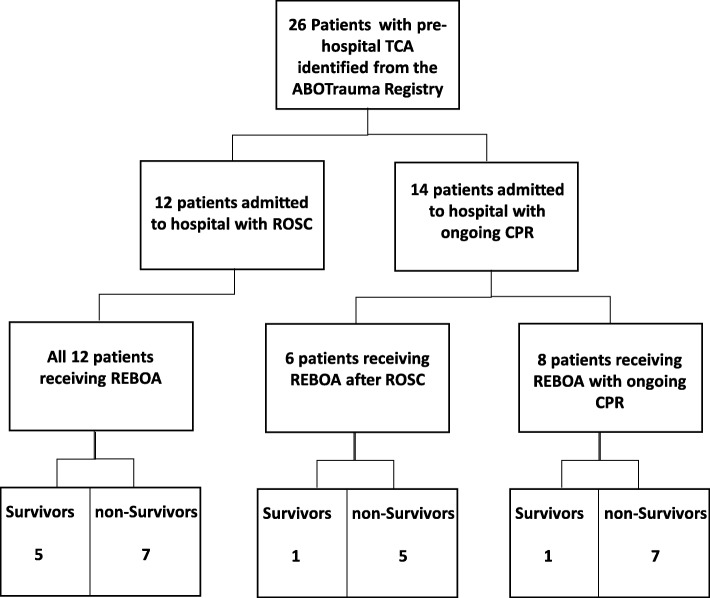
Flow chart visualizing who received REBOA post-ROSC vs. pre-ROSC. TCA traumatic cardiac arrest, ROSC return of spontaneous circulation

Of 213 patients reported in the ABOTrauma Registry during the study period, 26 patients (12.2%) who had received pre-hospital CPR due to TCA were identified. These 26 patients were treated at 11 centers in seven countries in Europe and Asia. None of the 26 patients received pre-hospital REBOA. The mechanism of injury in 21 patients (81%) was blunt trauma and in three patients (11.5%) penetrating trauma; data were missing for two patients (7.5%). The median (range) age of these 26 patients was 55 (8–79) years and 18 (69%) were male. Twelve patients (46%) had been admitted to the hospital with ROSC and 14 (54%) with ongoing CPR. Due to missing data, we cannot give a time range of the duration of the TCA. Of the 12 patients admitted to the ER with ROSC, five patients (42%) survived (see Fig. 1). In the group receiving ongoing CPR on admission, only two patients (14%) survived (see Fig. 1). Figure 1 provides a flow chart visualizing patients who received REBOA post-ROSC vs. pre-ROSC and the survivors in each group. The median (range) Injury Severity Score (ISS) was 45.5 (25–75) while the median (range) NISS was 46.5 (25–75). In all patients, data were sufficient to calculate the probability of survival using the RISC II. The RISC II predicted a mortality of 0.977, and therefore, the predicted mortality was 25 out of 26 patients (0.977 × 26 = 25.4 ≈ 25). Of the 26 patients, 17 (65%) survived post 24 h and seven patients (27%) survived to hospital discharge. Mortality following early REBOA after hospital admission (19 out of 26) was significantly lower (*p* = 0.049) than the predicted mortality (25 out of 26). Of the nine patients who died within the first 24 h, six died in the ER and three died during an emergency operation in the OR. Those who died after 24 h (*n* = 10) died after, median (range), 1.5 (1–6) days in the ICU. The SMR was 0.798, meaning that fewer patients died than predicted.

As expected, the investigated patient cohort had a pronounced trauma-induced coagulopathy (TIC) presented by a median (range) INR of 1.55 (1.08–9.96), aPTT of 66.8 (23.3–180) seconds and relatively low platelet count of 116.000 (12.000–335.000). Furthermore, the patients were in pronounced shock with a median (range) base excess of − 16.5 (minus 4.3–minus 28) and lactate of 11.7 (2.1–18.9) mmol/l.

## Transfusion requirements

The median (range) hemoglobin value on admission was 6.1 (2.4–8.8) mmol/l or 9.8 (3.8–14.2) g/dl. In 20 patients, transfusion was documented and, median (range), 22 (4–58) packed red blood cells (pRBC), 20 (6–70) fresh frozen plasma (FFP), and 5 (1–80) platelet packs were transfused within the first 24 h. In six patients, transfusion before aortic occlusion was documented. In these six patients, a median (range) of three (1-7) pRBC were given before REBOA, two out of the six patients received two FFP respectively and one patient received one platelet pack before aortic balloon occlusion.

## Special considerations regarding REBOA

**Fig. 2:**
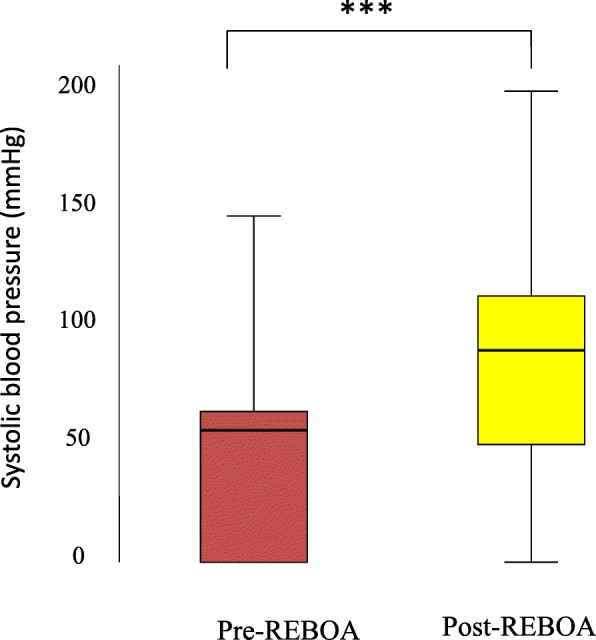
Boxplot of systolic blood pressure (mmHg) immediately before and after REBOA inflation for 26 patients who had pre-hospital CPR and early REBOA after hospital admission The interquartile range (IQR) is resembled by the box where it begins with the 25th percentile and ends with the 75th percentile. The median is represented as a line within the box. ***p = 0.001

Access to the common femoral artery was blindly achieved in 20 cases (76.5%) by ultrasound in three cases (11.5%) and by cut down in one case (4%) and was unknown in two cases (8%). Access was achieved by an emergency physician in 14 cases (54%), by a radiologist in four cases (15%), by a vascular surgeon and an anesthesiologist respectively in three cases (11.5%), and by a trauma and general surgeon in one case each (4%). REBOA resulted in a significant rise in systolic blood pressure (SBP), i.e., an increase in SBP ≥ 20 mmHg, as shown in Fig. 2.

Except for one patient, all survivors were responders to the initial REBOA attempt (87%), with a significant rise in SBP after aortic occlusion (see Table 1). After aortic occlusion, the median (range) SBP was raised from 57 (0–80) mmHg to 90 (0–136) mmHg in survivors (see Table 2). In patients who died, only 10 patients (53%) responded (increase in SBP ≥ 20 mmHg) to the initial REBOA attempt. In the group of non-survivors, the median (range) SBP was raised from 53 (0–147) mmHg to 90 (0–200) mmHg on average.

**Table 1:** Comparison between REBOA responders and non-responders

	Responders (*n* = 16)	Non-responders (*n* = 10)	*p* value
Age (years)	40 (8–79)	62.5 (20–78)	0.39
Male to female	11:5	7:3	0.99
Blunt to penetrating trauma*	13:3	8:0	0.53
ISS	51 (25–75)	42 (25–75)	0.52
NISS	51 (25–75)	43 (25–75)	0.62
SBP before REBOA (mmHg)	56.5 (0–80)	40 (0–147)	0.76
SBP after REBOA (mmHg)	95.5 (43–200)	40 (0–131)	0.03
partial-REBOA*	7/12 (58%)	3/6 (50%)	0.99
REBOA time (minutes)	45 (17–70)	28.5 (8–70)	0.49
RISC mortality	98.1 (57.4–100)	95.9 (63.2–99.8)	0.34
Survival	6/16 (37.5%)	1/10 (10%)	0.19

Data are presented as median (range) or number as appropriate. *p value* Mann-Whitney *U* test or Fisher’s exact test as appropriate

*ISS* Injury Severity Score, *NISS* New Injury Severity Score, *SBP* systolic blood pressure, *REBOA* resuscitative endovascular balloon occlusion of the aorta, *RISC* Revised Injury Severity Classification

*Numbers do not add up to 26 due to missing data

**Table 2:** Comparison between survivors and non-survivors

Variable	Survivors (*n* = 7)	Non-survivors (*n* = 19)	*p* value
Age (years)	40 (30–78)	60 (8–79)	0.69
Male to female	3:4	15:4	0.15
CPR on arrival**	2/6	12/17	0.16
Blunt to penetrating trauma**	7:0	14:3	0.53
Head injury	3/7 (43%)	10/19 (53%)	0.99
GCS at scene	3 (3–10)	3 (3–14)	0.66
ISS	41 (38–59)	50 (25–75)	0.78
NISS	41 (38–59)	50 (25–75)	0.4
RISC mortality (%)	94.3 (57.4–98.4)	98.5 (63.2–100)	0.03
REBOA zone I: zone III	6:1	15:4	0.99
REBOA responders	6 (86%)	10 (53%)	0.19
SBP before REBOA (mmHg)	57 (0–80)	53 (0–147)	0.88
SBP after REBOA (mmHg)	90 (0–136)	90.5 (0–200)	0.88
REBOA time (minutes)	45 (17–65)	35 (8–70)	0.68
pRBC*	24 (20–58)	15 (0–42)	0.3
FFP*	28 (6–70)	18 (0–38)	0.23
Platelets*	30 (0–80)	2 (0–30)	0.23

Data are presented as median (range) or number as appropriate. *p value* Mann-Whitney *U* test or Fisher’s exact test as appropriate

*GCS* Glasgow Coma Scale, *ISS* Injury Severity Score, *NISS* New Injury Severity Score, *RISC* Revised Injury Severity Classification, *REBOA* resuscitative endovascular balloon occlusion of the aorta, *SBP* systolic blood pressure, pRBC packed red blood cells, *FFP* fresh frozen plasma

*Values refer to documented cases with transfusion

**Numbers do not add up to 26 due to missing data

The median (range) duration of aortic occlusion was 45 (8–70) minutes, and there was no difference in occlusion time between survivors and non-survivors (*p* = 0.68). REBOA was deployed in zone I in 21 patients and in zone III in five patients. Three blunt trauma patients underwent resuscitative thoracotomy, performed by an EMS physician in the field, and they arrived at the hospital with ongoing CPR subsequently and died. One resuscitative thoracotomy (RT) was performed in the ER due to no measurable blood pressure followed by zone I REBOA. This patient was one of the survivors.

## Discussion

Our study has shown that the survival rate in patients having TCA and receiving REBOA early after hospital admission is significantly higher than predicted by the RISC II. Furthermore, REBOA significantly increases SBP. Patients responding to REBOA are more likely to survive compared with those not responding, although the results lack significance due to the low sample size.

Resuscitation of patients who sustain a TCA has been associated with a low rate of survival ranging between 0% and 35%, depending on the mechanism of injury [2, 6]. The high survival rate in our study (27%), despite the majority of patients being subjected to blunt trauma, justifies resuscitation efforts with the use of REBOA in patients with TCA.

Unfortunately, the ABOTrauma registry does not capture data regarding pre-hospital times, and therefore, we cannot estimate the length of TCA and CPR in our study. One could assume that REBOA in patients with a brief period of TCA is more beneficial than in patients with TCA for more than 30 min. The different survival rates, 42% in patients with ROSC at hospital admission vs 14% in patients with ongoing CPR, support this assumption.

Even in non-TCA, current studies assume a possible benefit for aortic balloon occlusion due to increased coronary and cerebral blood pressure [10, 11, 16]. The current evidence for the possible effects of REBOA during CPR is summarized in table S1. As one can see, there is evidence from animal studies that REBOA during CPR increases blood pressure, coronary, and cerebral perfusion with a slight increase in ROSC. Evidence from the human use date regarding these effects is much weaker and mainly based on case reports and small case series.

Current guidelines recommend to take RT into consideration in patients with TCA if CPR duration is less than 15 min [4]. The RT rate in our study is relatively low (11.5%), and we are unable to give a solid explanation for this. One possible explanation could be that the majority of patients included in this study presented with TCA after blunt trauma and RT in blunt trauma is disputed, since the survival rate is very low [6]. Another explanation could be a difference in local skills and algorithms, concerning the implementation of RT.

Massive bleeding is a preventable cause of death in trauma patients [17, 18]. In our study, 16 patients of the non-survivors died in less than 48 h after trauma with a median transfusion of 22 packed red blood cells making traumatic hemorrhagic shock and exsanguination the most plausible cause of death. The use of local compression, hemostatic agents, and adjunct tourniquets are often sufficient to stop or reduce external bleeding in the pre-hospital setting [19, 20]. Non-compressible torso hemorrhage in the abdomen or pelvis is difficult to control without surgery. REBOA may reduce bleeding below the occlusion zone, stabilizing the hemodynamics by increasing coronary and cerebral blood flow, and improving oxygenation of the heart and brain during CPR and is associated with a more favorable acid-base status of circulating blood [5]. More than 60% of our patients responded to REBOA with an increased SBP of more than 20 mmHg.

The RISC II predicted median survival rate of 2.2% in our patients is similar to other studies describing a survival rate of less than 8%, and the survival rate for TCA is still lower than in medical out-of-hospital cardiac arrest [21–27]. Interventions treating reversible causes of TCA may improve survival and neurological outcome. The survival rate in our study supports the use of REBOA in TCA. Access to the common femoral artery in our series was mainly blind. In contrast, other studies had a cut down rate of more than 50% [7, 28]. This can be explained by the fact that REBOA accesses were performed mainly by emergency physicians, radiologists, or anesthetists in our study.

Due to the necessary transfusion requirement, we assume the majority of our patients had massive bleeding and the application of early REBOA possibly prevented TCA. The median time of aortic occlusion in our study was 45 min in the patients who survived, 85% in zone I. Occlusion time up to 30 min in zone I is associated with a lower risk of complications [9, 29]. Nevertheless, the optimal occlusion time may depend on other factors such as the presence of collaterals or an associated hemorrhage proximal to the level of occlusion.

All of our patients had pre-hospital CPR, and pre-hospital REBOA may have been beneficial to some [30–32]. This may improve blood pressure, reduce bleeding below the level of occlusion, and prevent true cardiac arrest [30–32]. Nevertheless, it is important not to prolong ischemia time because it increases the risk of ischemia-reperfusion injury. Currently, no recommendation can be given regarding pre-hospital REBOA [33] since more evidence is required. The majority of our patients suffered from TIC which is multifactorial, including tissue injury and hypoperfusion [34]; this explains the high transfusion requirements in our patients despite the use of REBOA.

As mentioned earlier, CPR in the field is initiated when the patient is unresponsive and the carotid artery pulse cannot be palpated and this per se does not confirm true TCA. We cannot assure that all patients in our study had true TCA; however, the majority of patients included in the study came from countries with pre-hospital emergency physicians on duty in the EMS (Emergency Medical Service) and HEMS (Helicopter Emergency Medical Service) systems, so we believe that an emergency physician in the field makes it highly probably that the patients had true TCA.

## Limitations

The range of age and injuries make it quite difficult to interpret the data of our study, but on the other hand, the results represent “real-life” data of a wide range of patients with only 2 similarities (TCA with pre-hospital CPR and early REBOA after hospital admission). Therefore, we have to acknowledge that our study has several limitations. *First*, our data have been retrieved from a partially retrospective registry, and our sample size is small. Therefore, the results could be more of an association than a cause-effect relationship. *Second*, the RISC II score has certain limitations, with age and injury severity as strong negative predictors of survival. This may underestimate the probability of survival in patients with reversible causes of TCA. *Third*, there were some missing values regarding transfusion requirements compared with data needed to compute RISC II, which were complete. *Fourth*, the ABOTrauma Registry was designed to capture REBOA-specific data and not evaluate the individual use of the technique. Accordingly, indications for and the efficacy of REBOA use are diverse. Pre-hospital times are not captured in the registry, and therefore, we cannot estimate the length of the TCA and CPR. Fifth, the registry included patients who had REBOA deployed and established and not in those in whom REBOA failed and was not established; therefore, we do not have a control group of patients with TCA and non-REBOA. *Finally*, this is an international study with limited control on the inclusion and exclusion criteria and therefore have a risk of selection bias in the studied population.

## Conclusions

Our study has shown that mortality in patients suffering from TCA and receiving REBOA early after hospital admission is significantly lower than predicted by the RISC II. Early in-hospital REBOA may improve survival after TCA and pre-hospital CPR. These encouraging results should be followed by prospective studies with larger numbers to define the exact role of REBOA in these critically ill patients.

## Supplementary information

**Additional file 1.** Evidence of aortic balloon occlusion.

## Data Availability

The datasets generated and/or analyzed during the current study are not publicly available due to ownership by the Department of Cardiothoracic and Vascular Surgery, Faculty of Medicine and Health, Örebro University, Örebro, Sweden, but are available from the corresponding author on reasonable request.

## Abbreviations

ABO: Aortic balloon occlusion
AHA: American Heart Association
aPTT: Activated partial thromboplastin time
CPR: Cardio pulmonary resuscitation
*EMS*: *Emergency medical service*
ER: Emergency room
ERC: European Resuscitation Council
FFP: Fresh frozen plasma
GCS: Glasgow Coma Scale
*HEMS*: *Helicopter Emergency Medical Service*
INR: International normalized ratio
ISS: Injury Severity Score
NISS: New Injury Severity Score
REBOA: Resuscitative endovascular balloon occlusion of the aorta
RISC: Revised Injury Severity Classification
ROSC: Return of spontaneous circulation
rPBC: Red packed blood cells
RT: Resuscitative thoracotomy
SBP: Systolic blood pressure
SMR: Standardized mortality ratio
TCA: Traumatic cardiac arrest
TIC: Trauma-induced coagulopathy

## References

[1] Kehoe A, Smith JE, Edwards A, Yates D, Lecky F (2015). The changing face of major trauma in the UK. Emerg Med J..

[2] Harris T, Masud S, Lamond A, Abu-Habsa M (2015). Traumatic cardiac arrest: a unique approach. Eur J Emerg Med..

[3] McGreevy DT, Abu-Zidan FM, Sadeghi M, Pirouzram A, Toivola A, Skoog P, et al. Feasibility and clinical outcome of REBOA in patients with impending traumatic cardiac arrest. Shock. 2019. 10.1097/SHK.0000000000001500 31851119

[4] Truhlar A, Deakin CD, Soar J, Khalifa GE, Alfonzo A, Bierens JJ (2015). European Resuscitation Council Guidelines for Resuscitation 2015: Section 4. Cardiac arrest in special circumstances. Resuscitation..

[5] Burlew CC, Moore EE, Moore FA, Coimbra R, McIntyre RC, Davis JW (2012). Western Trauma Association critical decisions in trauma: resuscitative thoracotomy. J Trauma Acute Care Surg.

[6] Seamon MJ, Haut ER, Van Arendonk K, Barbosa RR, Chiu WC, Dente CJ (2015). An evidence-based approach to patient selection for emergency department thoracotomy: a practice management guideline from the Eastern Association for the Surgery of Trauma. The Journal of Trauma and Acute Care Surgery..

[7] Brenner M, Teeter W, Hoehn M, Pasley J, Hu P, Yang S (2018). Use of resuscitative endovascular balloon occlusion of the aorta for proximal aortic control in patients with severe hemorrhage and arrest. JAMA Surg..

[8] Truhlář A, Deakin CD, Soar J, Khalifa GEA, Alfonzo A, Bierens JJLM (2015). Kreislaufstillstand in besonderen Situationen. Notfall + Rettungsmedizin.

[9] Kulla M, Popp E, Knapp J (2019). Resuscitative endovascular balloon occlusion of the aorta: an option for noncompressible torso hemorrhage?. Curr Opin Anaesthesiol..

[10] Dogan EM, Beskow L, Calais F, Horer TM, Axelsson B, Nilsson KF (2019). Resuscitative endovascular balloon occlusion of the aorta in experimental cardiopulmonary resuscitation: aortic occlusion level matters. Shock..

[11] Daley J, Morrison JJ, Sather J, Hile L (2017). The role of resuscitative endovascular balloon occlusion of the aorta (REBOA) as an adjunct to ACLS in non-traumatic cardiac arrest. The American Journal of Emergency Medicine..

[12] Qasim Z, Brenner M, Menaker J, Scalea T (2015). Resuscitative endovascular balloon occlusion of the aorta. Resuscitation..

[13] Joseph B, Zeeshan M, Sakran JV, Hamidi M, Kulvatunyou N, Khan M, et al. Nationwide analysis of resuscitative endovascular balloon occlusion of the aorta in civilian trauma. JAMA Surg. 2019. 10.1001/jamasurg.2019.0096 PMC6584250 30892574

[14] Lefering R (2009). Development and validation of the Revised Injury Severity Classification (RISC) score for severely injured patients. Eur J Trauma Emerg Surg..

[15] Lefering R, Huber-Wagner S, Nienaber U, Maegele M, Bouillon B (2014). Update of the trauma risk adjustment model of the TraumaRegister DGU: the Revised Injury Severity Classification, version II. Crit Care..

[16] Brede JR, Lafrenz T, Klepstad P, Skjaerseth EA, Nordseth T, Sovik E (2019). Feasibility of pre-hospital resuscitative endovascular balloon occlusion of the aorta in non-traumatic out-of-hospital cardiac arrest. J Am Heart Assoc.

[17] Teixeira PG, Inaba K, Hadjizacharia P, Brown C, Salim A, Rhee P (2007). Preventable or potentially preventable mortality at a mature trauma center. J Trauma..

[18] Gruen RL, Jurkovich GJ, McIntyre LK, Foy HM, Maier RV (2006). Patterns of errors contributing to trauma mortality: lessons learned from 2,594 deaths. Ann Surg..

[19] Hilbert-Carius P, Wurmb T, Lier H, Fischer M, Helm M, Lott C (2017). Care for severely injured persons: update of the 2016 S3 guideline for the treatment of polytrauma and the severely injured. Der Anaesthesist..

[20] Spahn DR, Bouillon B, Cerny V, Duranteau J, Filipescu D, Hunt BJ (2019). The European guideline on management of major bleeding and coagulopathy following trauma: fifth edition. Crit Care..

[21] Engdahl J, Bang A, Karlson BW, Lindqvist J, Herlitz J (2003). Characteristics and outcome among patients suffering from out of hospital cardiac arrest of non-cardiac aetiology. Resuscitation..

[22] Grasner JT, Wnent J, Seewald S, Meybohm P, Fischer M, Paffrath T (2011). Cardiopulmonary resuscitation traumatic cardiac arrest--there are survivors. An analysis of two national emergency registries. Crit Care.

[23] David JS, Gueugniaud PY, Riou B, Pham E, Dubien PY, Goldstein P (2007). Does the prognosis of cardiac arrest differ in trauma patients?. Critical Care Medicine..

[24] Lockey D, Crewdson K, Davies G (2006). Traumatic cardiac arrest: who are the survivors?. Ann Emerg Med..

[25] Pickens JJ, Copass MK, Bulger EM (2005). Trauma patients receiving CPR: predictors of survival. J Trauma..

[26] Konesky KL, Guo WA (2018). Revisiting traumatic cardiac arrest: should CPR be initiated?. Eur J Trauma Emergency Surg.

[27] Escutnaire J, Genin M, Babykina E, Dumont C, Javaudin F, Baert V (2018). Traumatic cardiac arrest is associated with lower survival rate vs. medical cardiac arrest - results from the French national registry. Resuscitation.

[28] Brenner ML, Moore LJ, DuBose JJ, Tyson GH, McNutt MK, Albarado RP (2013). A clinical series of resuscitative endovascular balloon occlusion of the aorta for hemorrhage control and resuscitation. J Trauma Acute Care Surg.

[29] Knapp J, Bernhard M, Haltmeier T, Bieler D, Hossfeld B, Kulla M (2018). Resuscitative endovascular balloon occlusion of the aorta: option for incompressible trunk bleeding?. Der Anaesthesist..

[30] de Schoutheete JC, Fourneau I, Waroquier F, De Cupere L, O'Connor M, Van Cleynenbreugel K (2018). Three cases of resuscitative endovascular balloon occlusion of the aorta (REBOA) in austere pre-hospital environment-technical and methodological aspects. World J Emergency Surg.

[31] Lendrum R, Perkins Z, Chana M, Marsden M, Davenport R, Grier G (2019). Pre-hospital resuscitative endovascular balloon occlusion of the aorta (REBOA) for exsanguinating pelvic haemorrhage. Resuscitation..

[32] Manley JD, Mitchell BJ, DuBose JJ, Rasmussen TE. A modern case series of resuscitative endovascular balloon occlusion of the aorta (REBOA) in an out-of-hospital, combat casualty care setting. J Spec Oper Med. 17(1):1–8. 10.55460/9H3H-5GPS 28285473

[33] Borger van der Burg BLS, Kessel B, DuBose JJ, Horer TM, Hoencamp R. Consensus on resuscitative endovascular balloon occlusion of the aorta: a first consensus paper using a Delphi method. Injury. 2019. 10.1016/j.injury.2019.04.024 31047681

[34] Lier H, Bottiger BW, Hinkelbein J, Krep H, Bernhard M (2011). Coagulation management in multiple trauma: a systematic review. Intensive Care Med..

