# Supplementary material for: Pre-hospital CPR and early REBOA in trauma patients — results from the ABOTrauma Registry
Source: World J Emerg Surg. 2020 Mar 30;15:23. doi: 10.1186/s13017-020-00301-8 (PMC7104487; doi:10.1186/s13017-020-00301-8)
Supplement: Supplementary file 1 — Additional file 1. Evidence of aortic balloon occlusion. [file 13017_2020_301_MOESM1_ESM.docx]

**Table S1** Aortic balloon occlusion during CPR in traumatic cardiac arrest (TCA) and non-TCA. Feasibility and evidence of potential effects.

| ABO effect | **TCA Non-TCA**  Animal Studies | | **TCA Non-TCA**  Human use data | |
| --- | --- | --- | --- | --- |
| feasibility | ++ | ++ | + | + |
| Increased systolic blood pressure^1^ | ++ | ++ | + | + |
| Increased coronary perfusion pressure^1^ | ++ | ++ | + | + |
| Increased cerebral perfusion pressure^1^ | ++ | ++ | + | + |
| Bleeding control | ++ | n. a. | + | n.a. |
| Increased rate of ROSC | + | + / ++ | (+) | (+) |
| Survival benefit^2^ | n.a. | n.a. | (+) | (+) |

n.a. - not applicable

(+) possible benefit; + low evidence; ++ moderate evidence; +++ strong evidence

^1^ Increased blood and perfusion pressure are dependent of the ABO occlusion zone, proximal ZONE I occlusion is much more effective than ZONE III occlusion.

^2^ Long term survival is not investigated in animal studies.

The **Table S1** summarizes the possible evidence of aortic occlusion during CPR as described in the literature by animal studies and human data (1-28).

1. Abu-Nema T, Ayyash K, Berntman L, Matani Y, Sigurdsson GH, Francis I, et al. Intraaortic balloon tamponade during hemorrhagic shock in sheep. Circ Shock. 1988;24(1):55-62.

2. Abu-Zidan FM. Letter Regarding: Large Animal Models of Proximal Aortic Balloon Occlusion in Traumatic Hemorrhage. The Journal of surgical research. 2020;245:600-1.

3. Avyash K, Sigurdsson GH, Matani Y, Francis I, Abu-Nema T. Intermittent intra-aortic balloon tamponade during hemorrhagic shock. Microcirc Endothelium Lymphatics. 1988;4(6):419-31.

4. Barnard EBG, Manning JE, Smith JE, Rall JM, Cox JM, Ross JD. A comparison of Selective Aortic Arch Perfusion and Resuscitative Endovascular Balloon Occlusion of the Aorta for the management of hemorrhage-induced traumatic cardiac arrest: A translational model in large swine. PLoS medicine. 2017;14(7):e1002349.

5. Borger van der Burg BLS, Maayen R, van Dongen T, Gerben C, Eric C, DuBose JJ, et al. Feasibility Study Vascular Access and REBOA Placement: From Zero to Hero. Journal of special operations medicine : a peer reviewed journal for SOF medical professionals.18(4):70-4.

6. Brede JR, Lafrenz T, Klepstad P, Skjaerseth EA, Nordseth T, Sovik E, et al. Feasibility of Pre-Hospital Resuscitative Endovascular Balloon Occlusion of the Aorta in Non-Traumatic Out-of-Hospital Cardiac Arrest. Journal of the American Heart Association. 2019;8(22):e014394.

7. Daley J, Morrison JJ, Sather J, Hile L. The role of resuscitative endovascular balloon occlusion of the aorta (REBOA) as an adjunct to ACLS in non-traumatic cardiac arrest. The American journal of emergency medicine. 2017;35(5):731-6.

8. Deakin CD. Intra-aortic administration of epinephrine above aortic occlusion does not alter outcome of experimental cardiopulmonary resuscitation. Resuscitation. 2000;44(1):75.

9. Dogan EM, Beskow L, Calais F, Horer TM, Axelsson B, Nilsson KF. Resuscitative Endovascular Balloon Occlusion of the Aorta in Experimental Cardiopulmonary Resuscitation: Aortic Occlusion Level Matters. Shock. 2019;52(1):67-74.

10. Gedeborg R, Rubertsson S, Wiklund L. Improved haemodynamics and restoration of spontaneous circulation with constant aortic occlusion during experimental cardiopulmonary resuscitation. Resuscitation. 1999;40(3):171-80.

11. Gedeborg R, Silander HC, Rubertsson S, Wiklund L. Cerebral ischaemia in experimental cardiopulmonary resuscitation--comparison of epinephrine and aortic occlusion. Resuscitation. 2001;50(3):319-29.

12. Kauvar DS, Dubick MA, Martin MD. Response Regarding: Large Animal Models of Proximal Aortic Balloon Occlusion in Traumatic Hemorrhage. The Journal of surgical research. 2020;245:602-3.

13. Kauvar DS, Dubick MA, Martin MJ. Large Animal Models of Proximal Aortic Balloon Occlusion in Traumatic Hemorrhage: Review and Identification of Knowledge Gaps Relevant to Expanded Use. The Journal of surgical research. 2019;236:247-58.

14. Manning JE, Batson DN, Payne FB, Adam N, Murphy CA, Perretta SG, et al. Selective aortic arch perfusion during cardiac arrest: enhanced resuscitation using oxygenated perflubron emulsion, with and without aortic arch epinephrine. Annals of emergency medicine. 1997;29(5):580-7.

15. Manning JE, Murphy CA, Jr., Hertz CM, Perretta SG, Mueller RA, Norfleet EA. Selective aortic arch perfusion during cardiac arrest: a new resuscitation technique. Annals of emergency medicine. 1992;21(9):1058-65.

16. Nozari A, Rubertsson S, Wiklund L. Intra-aortic administration of epinephrine above an aortic balloon occlusion during experimental CPR does not further improve cerebral blood flow and oxygenation. Resuscitation. 2000;44(2):119-27.

17. Nozari A, Rubertsson S, Wiklund L. Improved cerebral blood supply and oxygenation by aortic balloon occlusion combined with intra-aortic vasopressin administration during experimental cardiopulmonary resuscitation. Acta anaesthesiologica Scandinavica. 2000;44(10):1209-19.

18. Reva VA, Horer TM, Makhnovskiy AI, Sokhranov MV, Samokhvalov IM, DuBose JJ. Field and en route resuscitative endovascular occlusion of the aorta: A feasible military reality? The journal of trauma and acute care surgery. 2017;83(1 Suppl 1):S170-s6.

19. Ross EM, Redman TT. Feasibility and Proposed Training Pathway for Austere Application of Resuscitative Balloon Occlusion of the Aorta. Journal of special operations medicine : a peer reviewed journal for SOF medical professionals. 2018;18(1):37-43.

20. Rubertsson S, Bircher NG, Alexander H. Effects of intra-aortic balloon occlusion on hemodynamics during, and survival after cardiopulmonary resuscitation in dogs. Critical care medicine. 1997;25(6):1003-9.

21. Ryan G, Swift K, Williamson F, Scriven E, Zheng O, Eley R. Feasibility of REBOA-Resuscitative Endovascular Balloon Occlusion of the Aorta-in Trauma-Related Noncompressible Torso Hemorrhage at Two Metropolitan Trauma Centers. The Ochsner journal. 2018;18(3):201-3.

22. Sesma J, Labandeira J, Sara MJ, Espila JL, Arteche A, Saez MJ. Effect of intra-aortic occlusion balloon in external thoracic compressions during CPR in pigs. The American journal of emergency medicine. 2002;20(5):453-62.

23. Spence PA, Lust RM, Chitwood WR, Jr., Iida H, Sun YS, Austin EH, 3rd. Transfemoral balloon aortic occlusion during open cardiopulmonary resuscitation improves myocardial and cerebral blood flow. The Journal of surgical research. 1990;49(3):217-21.

24. Teeter WA, Conti BM, Wasicek PJ, Morrison JJ, Parsell D, Gamble B, et al. Feasibility of basic transesophageal echocardiography in hemorrhagic shock: potential applications during resuscitative endovascular balloon occlusion of the aorta (REBOA). Cardiovascular ultrasound. 2018;16(1):12.

25. Tiba MH, McCracken BM, Cummings BC, Colmenero CI, Rygalski CJ, Hsu CH, et al. Use of resuscitative balloon occlusion of the aorta in a swine model of prolonged cardiac arrest. Resuscitation. 2019;140:106-12.

26. Coniglio C, Gamberini L, Lupi C, Cavallo P, Tartaglione M, Chiarini V, et al. Resuscitative Endovascular Balloon Occlusion of the Aorta for Refractory Out-of-Hospital Non-Traumatic Cardiac Arrest - A Case Report. Prehosp Disaster Med. 2019;34(5):566-8.

27. Aslanger E, Golcuk E, Oflaz H, Yilmaz A, Mercanoglu F, Bugra Z, et al. Intraaortic balloon occlusion during refractory cardiac arrest. A case report. Resuscitation. 2009;80(2):281-3.

28. Deakin CD, Barron DJ. Haemodynamic effects of descending aortic occlusion during cardiopulmonary resuscitation. Resuscitation. 1996;33(1):49-52.
